# Multifactorial Causation and Therapies of Myopia

**DOI:** 10.3390/jcm14092964

**Published:** 2025-04-25

**Authors:** César Villa-Collar, Cristina Álvarez-Peregrina, David P Piñero

**Affiliations:** 1 Department of Pharmacy, Biotechnology, Nutrition, Optics and Optometry, Faculty of Biomedical and Health Sciences, European University of Madrid, Villaviciosa de Odón, 28670 Madrid, Spain; 2 Department of Optometry and Vision, Faculty of Optics and Optometry, Universidad Complutense de Madrid, 28037 Madrid, Spain; cristina_alvarez@ucm.es; 3 Department of Optics, Pharmacology and Anatomy, University of Alicante, 03690 Alicante, Spain; david.pinyero@ua.es

Incidences of myopia have rapidly increased worldwide, particularly in children, along with the prevalence of high myopia [1]. However, it remains unclear whether cases of pathological myopia will increase alongside the rise in myopia itself. Furthermore, it has not been clarified whether the genes responsible for pathological myopia are the same as those for myopia in general, or if pathological myopia is genetically distinct from other forms of myopia [2]. In any case, it is currently believed that simply correcting refractive errors is no longer sufficient. Managing myopia with treatments developed for its control should not be optional, but rather, obligatory, for all eye care professionals. This Special Issue, titled “Multifactorial Causation and Therapies of Myopia”, aims to review the current context and future prospects of myopia, addressing its causes, current management, and potential control. Five works contribute to this collection, and the following lines contextualise these in relation to novel understandings of this topic.

Myopic choroidal neovascularisation (mCNV) is one of the leading causes of visual impairment and is associated with pathological myopia [3,4,5,6,7]. It occurs when abnormal choroidal neovascularisation penetrates the upper layers of the choroid, causing haemorrhage and exudation. Without intervention, this condition can severely impair central vision, significantly impacting patients’ quality of life [8]. This has driven research and innovation in therapeutic approaches to control and mitigate its effects. The first contribution, by Tomita et al. (Contribution 1), assesses the one-year results of intravitreal injections of the biosimilar ranibizumab (RBZ-BS) for mCNV in Japanese patients. The results show significant improvement in corrected visual acuity (BCVA) and reductions in central macular thickness (CMT), especially in treatment-naive patients. The authors conclude that the introduction of RBZ-BS is expected to alleviate the financial burden on patients and healthcare systems, while preserving high therapeutic efficacy, making it a valuable addition to the treatment of mCNV.

Undoubtedly, one of the most important objectives in this field is the prevention or delay of myopia onset and/or the deceleration of its progression. However, to achieve this, under standing aspects related to the profile that contributes to myopia onset and progression is essential. An example of one such aspectis the percentiles of various related topics, such as ocular biometry [9,10,11,12,13]. In Contribution 2, by Gopalakrishnan et al. (Contribution 2), the authors present the results of the Sankara Nethralaya Tamil Nadu Essilor (STEM) study, an ongoing longitudinal study involving approximately 14,000 children. The aim of the study is to understand the prevalence, incidence, and risk factors for the onset and progression of myopia in schoolchildren in southern India. Its objective is to investigate axial length percentile charts and axial length/corneal curvature (AL/CR) ratios, developed from baseline cross-sectional measurements of the STEM study, and to validate the usefulness of percentile measurements for identifying the risk of myopia development in these children based on longitudinal data.

The authors concluded that, when evaluating children at risk of myopia, close follow-up and early intervention could be applied to children with a higher percentile than the median (>50th), or those with an axial length of 23.30 mm or an AL/CR ratio of 3 or greater. Similarly, a shift in percentile at follow-up visits could indicate possible myopia development, and frequent follow-ups and timely intervention could be offered to such children.

On the topic of progress monitoring, one of the most widely used treatments worldwide is orthokeratology (OK) [14,15]. In 2023, OK lens fittings accounted for 1% of contact lens fittings worldwide. Contributions 3 and 4, by Martinez-Plaza et al. (Contribution 3) and Batres et al. (Contribution 4), address various aspects of this technique.

Specifically, Martinez-Plaza et al. focus on the compression factor (also known as the Jessen factor). After overnight contact lens (CL) wear and lens removal, the orthokeratologic effect regressed by approximately 0.50 to 0.75 dioptres (D) [16]. Orthokeratologic designs incorporated a compression factor that overcorrected the refractive error to compensate for this regression [17]. Traditionally, a conventional compression factor (CCF) of the same magnitude as the regression (0.50 to 0.75 D) is used. However, numerous studies have shown that this overcorrection is not always equivalent to the intended correction and, consequently, an increased compression factor (ICF) has been introduced in some orthokeratologic lenses [18,19,20]. This study evaluated the safety, efficacy, and visual performance of an orthokeratologic lens with a 1.25 D ICF during a three-month follow-up. The authors found that myopia correction with this ICF provided a good safety profile, efficacy, and visual performance.

Batres et al. evaluated OK lens centration, a key factor of successful fitting. To achieve satisfactory optical results, the treatment zone (TZ) was centred on the cornea. The estimated shift between the pupillary centre and the nominal centre of the TZ indicated the off-centre position of the lens fitting, a frequent and unavoidable phenomenon, even with an optimal fitting. Complications resulting from off-centre lenses provoked reduced contrast sensitivity, causing symptoms such as halos, glare, and blurred vision [21,22]. The objective of this study was to examine the trend in off-centre TZ during 12 months of OK lens use using two Corneal Refractive Therapy (CRT) lens designs: standard (STD) and dual-axis (DA).

The authors concluded that off-centre lens design was similar in both STD and DA after one year of follow-up. The spherical design tended to be off-centre horizontally during the first 6 months, while the toric design reached the maximum vertical off-centre position at the end of the year.

The final contribution (Contribution 5) addresses a critical aspect of research in the pathophysiology of myopia. In their review article, Baksh et al. summarised changes in choroidal thickness related to variations in choroidal flow (CF), the association between near work and myopia, and choroidal structural changes caused by accommodation. The choroid thickens in response to myopic defocus and thins with hyperopic defocus. Pharmacological interventions with antihypoxic drugs and vasodilators have been effective in inhibiting the development of myopia by altering CF. Furthermore, they emphasise spending time outdoors can prevent myopia, with studies showing a relationship between this and increased CF.

To improve our understanding of the pathogenesis of myopia, more research is needed, specifically that focusing on choroidal changes, as this holds great potential [23,24]. Such investigations can substantially contribute to the identification of new therapeutic targets or to finding promising strategies for prevention.
